# The Power of Students: Using Positioning Theory and Frame Analysis to Explore Power Dynamics in Mentoring Relationships

**DOI:** 10.5334/pme.1662

**Published:** 2025-05-28

**Authors:** Hannelore van der Kloot, Erik Driessen, Eline Vanassche

**Affiliations:** 1 Faculty of Psychology and Educational Sciences, KU Leuven Kulak, Kortrijk, Belgium; 2 Medical Education at the Faculty of Health, Medicine and Life Sciences, Maastricht University, Maastricht, the Netherlands

## Abstract

**Introduction:**

Power plays a crucial role in mentoring relationships as mentoring is inherently a social and relational practice. Power dynamics can function as a gatekeeper to students’ learning opportunities and experiences, making it crucial to build a better theoretical and empirical understanding of how it operates in and through mentoring relationships. This study explores how students participate in power dynamics in mentoring relationships during their internship. It starts from the view that power is not solely tied to hierarchy, but is in a continuous flux, recognizing that students also participate in power dynamics and shape mentoring relationships.

**Methods:**

The study draws on a multiple case-study of four mentoring dyads in undergraduate general practice internships. Non-participant observations were combined with periodic interviews and audio diaries. We took a discursive perspective on power dynamics that was operationalized through the integration of positioning theory and frame analysis.

**Results:**

The analysis resulted in four enactments of power by students: resistance, imposed power, empowerment, and vested power. The four enactments can be situated on two axes, that is, doing power versus being enabled to do power; and whether the mentors’ and students’ frames align or not.

**Discussion:**

The findings documented the potential power of the students by introducing a novel discursive framework. Students enact power by doing power themselves or being enabled to do power by their mentors; supporting that students also have responsibilities in the mentoring relationship. Making students’ and mentors’ expectations and goals explicit can provide insight in supporting students during mentoring experiences.

## Introduction

Power plays a crucial role in mentoring relationships as mentoring is inherently a social and relational practice [1]. Mentors are typically more experienced and are asked to serve as mentors because of their extensive professional expertise [2]. Often, mentors are also clinical teachers and possible future colleagues of students. This makes mentoring relationships inherently hierarchical. However, power in mentoring relationships cannot be solely understood from the higher professional status of the mentor. Students themselves bring their own beliefs, expectations, and goals to the relationship, which can either conflict or align with those of the mentor [3]. This means students – although often viewed as powerless – are also influencing actors in the experiences and consequences of the mentoring relationship and therefore can participate in the power dynamics.

This study moves beyond the idea of power as related to a hierarchy in experience and expertise. We build on Foucault’s idea that the “institutional model” of power should make place for the so-called “strategic model” of power [4]. The former underscores the vertical relationship between actors and views power as embedded within formal structures and institutions. The latter model views power as a network of relationships and practices that operate fluidly and is exercised through social interactions and tactics. The strategic model shifts the focus of power from something that is connected to or even possessed by one actor, to something that is “de-faced” [5], and that occurs everywhere and anytime, in a continuous dynamic “flux” [4]. This conceptualization of “power as flux” from Foucault helps us to understand that power is not solely “owned” or “exercised” by the mentor. Rather, mentors as well as students can participate in power dynamics within the mentoring relationship.

Uncovering power dynamics, and students’ participation in them, can help to better understand the complex interactions of mentoring relationships that create or constrain opportunities to learn [6]. While such powerful and hierarchical relationships can benefit students’ professional development and career trajectories [7], they can also cause harm [8]. Harm refers to students experiencing inappropriate behavior such as bullying [9] or sexual harassment [10]. Potentially more common, but less discussed in the literature, are the more subtle tensions or conflicts experienced by students. For instance, they may feel that “they are not receiving proper credit for their ideas” [11, p. 94] or they do not learn what they wished for. These instances can lead to student dissatisfaction or missed learning opportunities [6].

The notion of “power as flux” can provide an analytic lens to better understand the complex interactions within mentoring and build a better theoretical and empirical understanding of how mentoring works. However, the fact that students can participate in power dynamics in mentoring relationships is rarely mentioned in the literature [notable exceptions are 3,12,13,14,15]. This article addresses this gap by exploring the research question: How do students participate in power dynamics in the mentor-student relationship during an internship? This study adds to existing research by focusing on the potential power of students within mentoring relationships, and by providing a conceptual-analytical framework based on positioning theory and frame analysis to study these power dynamics.

## Methods

### Study design

We conducted a multiple case-study of four mentoring dyads in undergraduate medical education [16]. The selection of four cases prioritizes depth over breadth, allowing for a detailed analysis of the complexity and dynamics of longitudinal mentoring relationships. Data collection combined non-participatory observations, audio diaries and interviews with students and mentors in each mentoring dyad to longitudinally study power dynamics in their context [17]. We started our study from a discursive perspective [18], focusing on the performativity of language (verbal and non-verbal). This perspective of performativity allows us to study power as “flux”, created in interaction, shaped by what is said and left unsaid, by whom and with what [19].

### Conceptual-analytical framework

We constructed a framework based on an integration of positioning theory and frame analysis to study the power dynamics. Both theories start from a discursive perspective [20] and acknowledge how interactions are mediated by relationships of power [21].

Central to positioning theory is the idea that actors are continuously positioning themselves and others in interaction [22]. This process is based on assigning or denying specific rights and duties; therefore, it is never neutral [23]. The positioning process reflects how actors discursively participate in power. For instance, the mentor denies the student the right to participate in patient care, and therefore the mentor positions the student as the observer of professional practice. This positioning shows that both actors participate in the power dynamics. The meaning of this positioning in terms of power depends on the expectations and goals of both actors. If the mentor positions the student because s/he is convinced that the student is not ready for safe patient care, the meaning of the power dynamics differs from when the student had explicitly asked to be an observer.

To gain insight in the expectations and goals of the actors, and thus to give additional meaning to power, we incorporate the concept of “frames” from frame analysis. In this study, frames represent the different expectations and goals of a particular actor toward the internship, influencing and directing the rights and duties for the actors involved within that practice. On the one hand, these frames are constructed from prior experiences, own beliefs and social structures within society [24]. On the other hand, these expectations and goals are “continually checked against experience and revised” in interaction [25, p. 207] and thus inherently dynamic.

We used the combination of positioning theory and frame analysis as a conceptual-analytical lens to explore how students discursively negotiate power in mentoring relationships.

### Setting, participants and recruitment

The multiple case-study consisted of four mentoring dyads. Mentoring in this study refers to the relationship between undergraduate students and their mentors during an internship as part of their initial training. The undergraduate students involved in this study were doing their final six-week internship in general practice in Spring 2023. General practice offers a unique clinical setting, smaller than hospital workplaces and therefore allowing more emphasis on the interpersonal interaction between mentor and student [26]. All participating students specifically opted to complete their final internship in general practice. During the internship, the students were supervised by a general practitioner who volunteered for the role of mentor. Although general practitioners are not formally required to follow a faculty development program to prepare them for their mentoring role in this specific internship, three out of four mentors did. All four mentors had ample experience with mentoring in these internships. Table 1 shows the profile of the mentors, together with the gender of both mentors and students. More detailed information about the participants cannot be provided to secure the participants’ confidentiality.

**Table 1:** Overview participants.


	DYAD 1	DYAD 2	DYAD 3	DYAD 4

**Mentor**	Man	Woman	Man	Man

	Experienced	Experienced	Experienced	Experienced

	Faculty development program	Faculty development program	No faculty development program	Faculty development program

**Student**	Woman	Man	Man	Man


The participants were recruited after ethical approval from the Ethics Committee Research of the university (S67273). Emails were sent on behalf of the internship office to general practitioners mentoring students in the final internship. The mentors notified the research team if they were interested. The mentors asked all respective students from the upcoming internship period if they were interested in participating. Students who showed interest were referred to the research team to receive the full information of what participation would evolve. The students gave their informed consent voluntarily, but we were aware of the hierarchical relationship between mentors and students, and students potentially feeling obligated to participate. However, students’ (and mentors’) consents were not considered a one-off challenge prior to the data collection, but rather an ongoing dialogue, negotiation and building trust during fieldwork.

### Data collection

**Figure 1:**
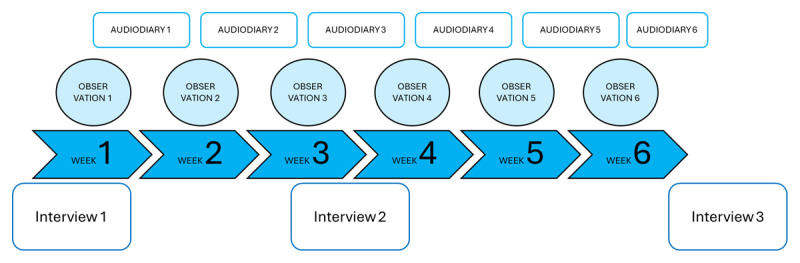
Overview Data Collection.

Weekly non-participatory observations [27] were central to data collection. We observed each dyad for a minimum of six half-days, resulting in a total of 200 hours of observation (see Figure 1). We observed interactions both during clinical practice (consultations at the general practice center and home visits to patients) as well as other formal and informal interactions, including feedback meetings and lunch breaks. The observations were supported through extensive field note writing, documenting verbal and non-verbal language use, the use of artifacts (e.g., doctor’s coat, chair or stool, gloves, cellphones) and important contextual information (e.g., layout of the cabinet) [28]. Participants were further invited to make sense of their experiences by asking them to document and reflect on critical incidents in an audio diary shared with the researcher on a weekly basis [29] (see Appendix 1). Preliminary understandings and reflections derived from the observations and audio diaries, were discussed in three periodic (beginning-middle-end) individual interviews with mentors and students [30] (see Appendix 2–3). Individual interviews were chosen to curb potential power dynamics and let the participants speak openly about moments that happened during the internship. These periodic interviews also served to build a deeper understanding of mentors’ and students’ evolving expectations and goals during particular moments of mentoring practice. This resulted in a total of 24 interviews that lasted on average one hour.

### Data analysis

**Figure 2:**
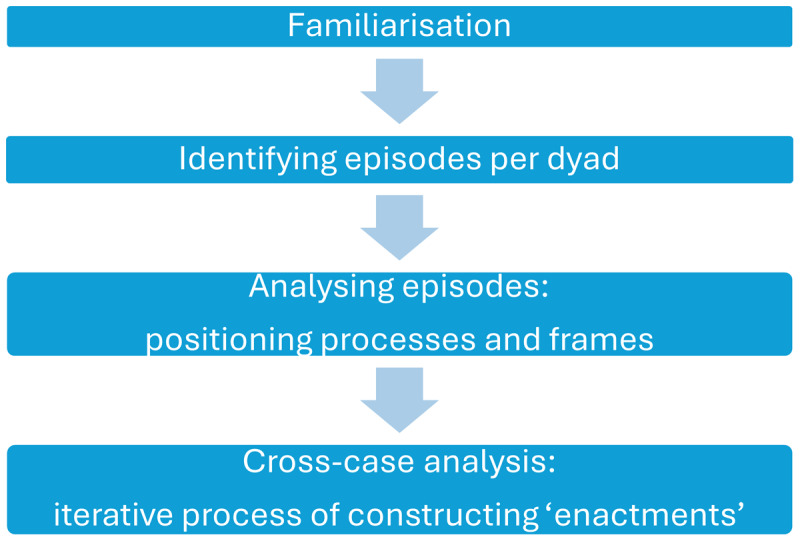
Overview data analysis.

Figure 2 shows that the analysis started with the first author familiarising herself with the data through intensive reading of the full dataset for each dyad [16]. In this process, memos were written as a “documentation of the researcher’s thinking processes” [28, p. 68] to get a first understanding of the power dynamics in the mentoring relationships. After familiarization, the analysis continued with a focus on the field notes. In the field notes, ‘episodes’, or sequences of interaction, were inductively and interpretatively identified that seemed significant for the mentoring relationship and might be indicative of (shifting) power dynamics (e.g., because of a change of subject, tone, modality, interaction pattern, etc.) [31–32]. These episodes were analyzed further from a positioning theory perspective, focusing on the way students were positioning themselves or being positioned. These positioning processes ‘produce’ the mentoring relationship and discursively attribute power. For instance, students assigned themselves the right to suggest an alternative advice for patients or mentors gave students the right to take the lead in performing a patient consultation. Second, we looked at how participants discursively framed the internship and the developing mentoring relationship to better understand the situation within which rights and duties were attributed and power was enacted. At this stage in the analysis, we included the interviews which documented participants’ evolving framing of the internship in general and, where possible, the specific episodes selected in the first stage of the analysis. For example, one student (dyad 1) expressed the goal to build confidence by independently seeing patients in the first interview and actively revisits and refines this goal in the interview following an observation where she actually performed a consultation on her own. Thus, these periodic interviews functioned both as an attempt to capture expectations and goals, as well as a communicative validation of occurrences during the internship [33].

Through this iterative process, a total of 30 to 45 episodes per dyad were retained in which students engaged in some form of power. These episodes were documented and extensively described in an analytical table that provided the basis for a cross-case analysis with the goal of clustering episodes based on the nature of power enacted by students [32]. Through constant comparison of episodes within and across dyads, and analytic dialogue with the research team, we ultimately identified and described four distinguishable forms of power enacted by students in mentoring relationships, described further below.

## Results

**Figure 3:**
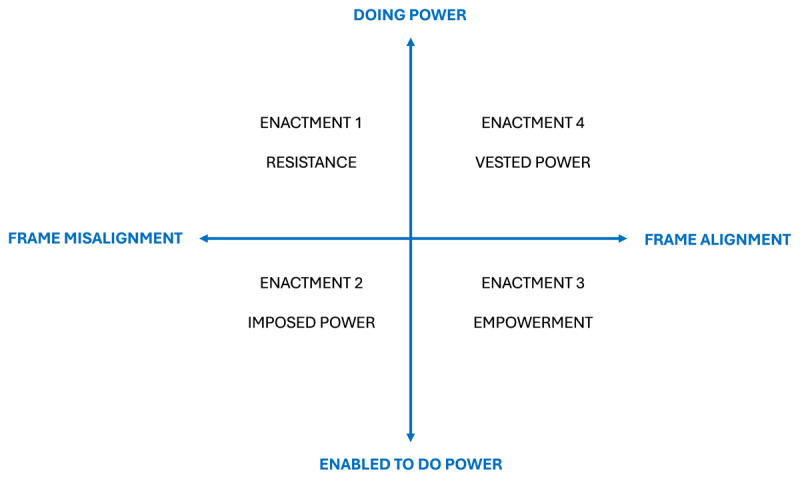
Four enactments of power.

The analysis resulted in four enactments of power by students, with mentors, during mentoring relationships: resistance, imposed power, empowerment and vested power. These types can be situated on two axes (see Figure 3). The first axis distinguishes *doing power* from *being enabled to do power*, based on the positioning processes at play. The distinction implies that when students are doing power, the students are positioning themselves in a position of power. When students are enabled to do power, it is the mentor who puts students in a position of power. We use the verb “to do”, rather than “to exercise” or “to own”, to highlight the discursive and dynamic process. The second axis distinguishes whether the frames of the students and mentors align or misalign during the positioning process.

All enactments of power occur in each dyad and continuously alternate, along with moments when mentors do power. They emerge both during patient consultations and home visits, as well as in informal interactions over lunchtime or formal mentoring meetings. An enactment of power may be present temporarily or persist for an extended period.

In the presentation of the findings, we purposefully use field note excerpts from the same dyad (dyad 3) to illustrate all four enactments of power. Focusing on one dyad allows us to provide in-depth information on how the mentor and student frame the situations during the mentoring practice, and thus their evolving expectations and goals for the internship, while respecting the journal’s wordcount. When describing excerpts of dyad 3, we use him/his terminology as the dyad consisted of two men: Victor (student) and Rick (mentor).^1^

### Resistance

The first enactment of power, resistance, occurs when students challenge and counterbalance the position of the mentors who are doing power within the relationship. Students position themselves in a position of power, assigning themselves rights and duties and acting according to their current expectations and goals for the internship (i.e. frames). Typical of resistance, is that the students’ goals and expectations do not align with those of the mentors during the ongoing situation, apparent in a misalignment of frames and associated power dynamics.

Excerpt 1 – Dyad 3 (Day 3, 9.15 AM)

After a visit to a patient in an elderly care home, the mentor consults with the nurse from the elderly care home about the policy for the patient. The student stands aside, says nothing, and looks at his cell phone.

By looking at his cellphone, Victor positions himself as a bystander of the conversation between the mentor and the nurse. Discussing his cellphone use during the internship in the final interview, Victor adds additional meaning to his positioning: “No, it wasn’t a signal, it was more out of boredom ((laughs))” (Interview 3 student). He expects to perform consultations, rather than doing home visits. Although the student does not necessarily intend to ‘resist the mentor’, Rick clearly noticed the student’s use of the cellphone, stating in an interview: “*In the car or even when we have to do a home visit, it’s noticeable that Victor is very busy with his phone. I’d prefer he didn’t do that, but then I think: ‘Maybe it’s important to him, I’m not going to say anything about it*.” (Interview 2 mentor). The act of Victor seems to have an effect on the mentor (see also excerpt 2) and thus this indicates how the student is enacting power.

Resistance thus occurs when students are doing power (positioning themselves) according to how they frame the situation. This framing is not aligned with the framing of the mentors. Resistance can be visible in a certain disinterest as in excerpt 1, but also as a disagreement, visible in enactments in other dyads.

As within this excerpt, an enactment of power can occur by using artefacts, nonverbal language or no direct interaction at all, next to using verbal language. This highlights both the subtility of power dynamics as well as the importance of taking into account nonverbal language and embodiment.

### Imposed power

In the second enactment of power, there is a second instance of misaligned frames. The difference with resistance is that mentors now put students in a power position, with the students appearing to be in charge within the mentoring practice. However, as this position is not according to the students’ frame(s) in use, but rather that of the mentor, power seems to be imposed upon students. This is shown in an excerpt that follows directly on excerpt 1, after the student took his cell phone during the discussion of a patient with the nursing staff of the elderly care home.

Excerpt 2 – Dyad 3 (Day 3, 9.30 AM)

The mentor asks the student at the next home visit: “Are you going to take charge?” The student hesitantly answers: “Humm, yes.” Afterwards, the student leads the home visit.

The mentor puts the student in a power position by asking him to lead the home visit. The student is now the active performer of the home visit. However, the student does not necessarily expect to be in charge of or engaged with home visits: *“The only annoying thing about this [internship] compared to the previous one is that it contains a lot of home visits; so you don’t have much opportunities to really work clinically, and think or reason, and actually have a normal consultation*” (Interview 1 student). Nevertheless, the mentor does not exactly know the frame of the student and therefore seems to believe he is supporting the student by actively engaging him.

Imposed power thus occurs when students are enabled to do power (being positioned by the mentor) while this is not aligned with the students’ frames.

### Empowerment

Empowerment means that mentors again enable the students’ power, as with imposed power. The difference between both enactments is that the students accept the mentors’ positioning of them, as it aligns with and therefore supports their own expectations and goals for the internship.

Excerpt 3 – Dyad 3 (Day 2, 12 AM)

On the first observation day, two patients arrive at the same time. The mentor asks the student which patient, and more specifically which case, he wants to treat. The student chooses a patient with pain in his shoulder, because, as he states, it is “the easiest”.

By allowing Victor to choose the next clinical case, the mentor enables the student to do power within the mentoring practice. The student chooses a case he is familiar with. He describes in the first interview: *“I think that by the end of this internship, I just want to have the confidence that I can welcome a patient in and send them away with a positive feeling.”* (Interview 1 student). By letting the student choose, the mentor seems to empower the student to act according to this student’s expectation for the internship. This excerpt also highlights that multiple frames are active in the course of a mentoring relationship, evolving from one interaction to another and potentially contradicting one another.

Thus, empowerment means that mentors are enabling the students’ power position by giving them a chance to act according to their frame and therefore supporting the ongoing expectations and goals of the student. As a result, the mentors’ actions support and empower the students’ expectations and goals.

### Vested power

Lastly, vested power occurs when students put themselves in a power position and this position is accepted by the mentors. Mentors align with, and therefore support and affirm, the active frame(s) of the students, in contrast to resistance.

Excerpt 4 – Dyad 3 (Day 4, 8 AM)

On the fourth observation day, the first consultation starts at 8 AM. The student has not arrived yet, so the mentor starts ahead. A few minutes later, the student enters the consultation room, to which the mentor comments: “Oy, didn’t sleep well?”. The student smiles but does not answer and goes directly to the patient to draw blood. The mentor asks if the student will draw blood, the student nods in agreement. Then the student draws blood and takes a seat in the main chair afterwards to continue the consultation. Meanwhile, the mentor fetches an extra stool, and places himself on the stool next to the student.

The student positions himself as the performer of the consultation, and albeit temporarily the “decision maker” of the clinical practice. The mentor accepts this position by letting him draw blood, by literally positioning himself on the stool next to him, and by not verbally questioning this situation afterwards. By taking initiative to draw blood and taking the seat in the main chair, the student seems to expect to perform the consultation himself. The mentor aligns with this situation and this framing. One of his goals for the internship, mentioned in an interview, is to offer the student as many learning opportunities as he can. Therefore, he expects the student to ask questions and take initiative. Nevertheless, it contradicts his expectation that the student shows interest and engagement during the internship, which also implies arriving on time.

As a result of vested power, students seem more likely to act dominant in the mentoring practice and rather disconnected from mentors. Students’ frames are foregrounded, often resulting in placing some of the frames of the mentors in the background.

## Discussion

We explored the ways in which students participate in power dynamics within mentoring relationships through a novel discursive framework that integrates positioning theory and frame analysis. The power dynamics could be clustered into four enactments of power by the student, that can be situated on two axes: doing power versus being enabled to do power, and whether mentors’ and students’ frames align or not. Our findings underpin that students actively engage in power dynamics by influencing and shaping the mentoring relationship in different, complex ways [14].

### Power as flux

The different enactments of power allow us to understand that power should not be seen as exclusively hierarchical and institutional but power operates more in a dynamic flux [4]. Moreover, the findings illustrate that and how students participate in the negotiation of truth claims [34] during their internship. The findings elaborate on the notion of “powerless students”, by introducing other enactments of power such as imposed power, vested power and empowerment; and therefore, extend beyond merely “navigating” or “resisting” mentors’ hierarchical power, often documented in the literature [e.g., 35–36]. This is in line with the findings of Lorentzen (2008) that mentoring relationships in medical education are “relational and productive, not merely repressive” power relations, and “constituted through the changing alignment and negotiated practices of individuals” [37, p. 53].

In all dyads, the four enactments of power by students continuously alternate, as do moments when mentors participate in power. Analyzing the temporal patterns of the enactments was not a primary focus in the analysis but there are indications that the enactments did not occur in random order. For example, imposed power would often follow resistance, as observed in excerpts 1 and 2. We invite future research to explore the sequence of the enactments (i.e., one power exchange serving as a precursor or precondition for another) as this could provide further insight into how mentoring relationships unfold and develop. Moreover, future research could explore if the enactments of power belong to particular stages of the internship (i.e., beginning, middle, end) as this again helps to understand their meaning in and for the mentoring relationship.

The findings allow an initial insight into the discursive multimodalities that students (and mentors) use to enact power. For example, the excerpt of resistance and vested power illustrated that the student used an artifact or nonverbal language rather than verbal communication when doing power. This highlights the importance of fully embracing the discursive perspective and understanding how verbal, nonverbal, and paraverbal communication can construct power [13,38]. Students and their mentors could enhance their awareness of the different multimodalities used to enact power [13] to deepen their understanding of the mentoring relationship’s dynamics.

### Power of students in mentoring relationships

Next to the dynamical and discursive nature of power, the enactments of power by students also shed light on the complexity of mentoring relationships and the resulting learning opportunities for students.

To better understand the complexity of mentoring relationships, we suggest increasing awareness among both students and mentors about positioning processes and their frames. First, positioning processes highlight that students can do power themselves, or they can be encouraged by mentors to take on positions of power. Second, and equally important, is making expectations and goals explicit. By having insight into how students frame particular situations (e.g., home visits, consultations etc.) in view of their learning and development (as part of a particular internship and their broader development as a junior clinician), mentors could provide better support. Similarly, reflecting on mentors’ frames can help students understand their – potentially well-intentioned – actions, even when they seem illogical from students’ perspectives. Aligning mentors’ and students’ frames can strengthen the mentoring relationship [39]. However, raising awareness of and defining frames is challenging, as frames are dynamic and emerge through interaction [40]. Video recordings of mentors’ and students’ interactions could for example help by enabling in-depth discussions of specific situations, allowing both mentors and students to clarify the intentions behind their actions [25].

Even when frames align, we should be careful to claim this necessarily leads to more “productive” or “better” learning opportunities for students. For example, excerpt 3 illustrated that the student was “empowered” by the mentor to choose the easiest consultation. In this situation, the student was given the right to build his confidence in performing consultations. At the same time, this may have prevented him from being pushed to perform a difficult consultation and thus actively pursue other learning opportunities. This illustrates how power is intricately intertwined with learning and that no single enactment of power is directly linked to more, fewer, better, or worse learning opportunities.

### Limitations

Our decision to sample mentoring relationships in general practice may explain *that* and *how* students participate in power dynamics, due to the intensive relationship between students and general practitioners. Also, the setting of the Western European context may provide an explanation for the power of students, as independence and taking initiative are important values. We acknowledge that the identified enactments may not exhaustively capture all enactments of power of students and that the current dataset may not provide access to other enactments of power. While we have continuously negotiated consent with students throughout the fieldwork, the initial recruitment of students via the mentor has limitations, particularly given our focus on power dynamics in mentoring relationships. Moreover, while not recording the observations was deliberate for patient safety, it also provides limited insight into exact (non/para) verbal communication among the enactments of power, since not all details of communication could be written down in the field notes.

## Conclusion

Although power is an important aspect of mentoring relationships, it is primarily viewed in terms of the mentors’ power over the student given their higher professional status. This study focuses on how students can participate in power dynamics within the mentoring relationship, namely through doing resistance, imposed power, empowerment and vested power. These findings illustrate the potential of students as responsible, influencing actors within mentoring relationships, and provide a novel framework to study power dynamics.

## Data Accessibility Statement

All data collection instruments were translated from Dutch to English.

## Additional Files

The additional files for this article can be found as follows:

10.5334/pme.1662.s1 Supplementary file 1. Appendix 1. Audio diary.

10.5334/pme.1662.s2 Supplementary file 2. Appendix 2. Interview guides for mentors.

10.5334/pme.1662.s3 Supplementary file 3. Appendix 3. Interview guides for students.

## Footnotes

All names are pseudonyms.
