# Supplementary material for: The Power of Students: Using Positioning Theory and Frame Analysis to Explore Power Dynamics in Mentoring Relationships
Source: Perspect Med Educ. 2025 May 28;14(1):328–38. doi: 10.5334/pme.1662 (PMC12124276; doi:10.5334/pme.1662)
Supplement: Supplementary file 1. — Appendix 1. Audio diary. [file pme-14-1-1662-s1.pdf]

AUDIO DIARY STUDENT

Purpose: The audio diaries aim to provide insights into meaningful interactions between the mentor and the student during moments when the researcher is not present

Timing: At least one audio diary should be submitted each week of the internship, totaling six audio diaries throughout the internship period.

CONTENT

Describe a critical incident from this internship week. Specifically, I'm interested in an event or situation that was in some way significant to your relationship with the mentor. The selection of the critical incident is entirely up to you, based on how you interpret and feel about certain situations. If multiple meaningful situations occur, feel free to share them all.

When documenting and exploring the event/situation, the following questions (based on Krings & Goedhuys, 2011) may serve as a helpful guide:

- Describe the situation: Where were you? When did it take place?
- Core task: What was the situation about?
- Behavior: What was said/done, and by whom?
- What did you say/do?
- How did the mentor respond to this?
- Outcome: What do you think the impact of this situation was—on you and on the mentor?

There is no need to focus on phrasing or structuring your story during the recording. Speak as if you were debriefing with a family member or friend.
The purpose of the audio diary is to give the researcher sufficient understanding of how the internship and your interactions with the mentor are progressing.

INSTRUCTIONS FOR RECORDING

- Download the app: Voice Record Pro.
- Select 'REC'.
    - A quality overview will appear; no adjustments are necessary.
    - Select 'REC' again.
- Start recording:
    - Begin by stating your name, the day, and the time of the recording. This helps me locate the fragment later.
    - Document and explore the event/situation you want to share (see guiding questions above).
- Stop the recording:
    - Select 'STOP'.
- Rename the file: Rename the file to "Location – Date – Student".
    - If you want to re-record, repeat the steps above.
- Send the recording:
    - Choose 'send by email'.
    - Select 'audio' and then 'email attachment'.
    - Send to hannelore.vanderkloot@kuleuven.be.
    - If the file is too large, choose 'download link'.

AUDIO DIARY MENTOR

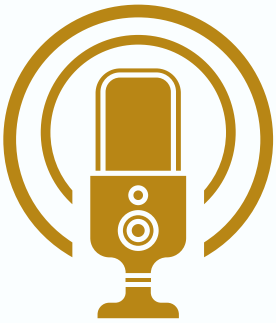

Purpose: The audio diaries aim to provide insights into meaningful interactions between the mentor and the student during moments when the researcher is not present.

Timing:
At least one audio diary should be submitted each week of the internship, totaling six audio diaries throughout the internship period.

CONTENT

Describe a critical incident from this internship week. Specifically, I'm interested in an event or situation that was in some way significant to your relationship with the student. The selection of the critical incident is entirely up to you, based on how you interpret and feel about certain situations.
If multiple meaningful situations occur, feel free to share them all.

When documenting and exploring the event/situation, the following questions (based on Krings & Goedhuys, 2011) may serve as a helpful guide:

- Describe the situation: Where were you? When did it take place?
- Core task: What was the situation about?
- Behavior: What was said/done, and by whom?
- What did you say/do?
- How did the student respond to this?
- Outcome: What do you think the impact of this situation was —on you and on the student?

There is no need to focus on phrasing or structuring your story during the recording. Speak as if you were debriefing with a family member or friend.
The purpose of the audio diary is to give the researcher sufficient understanding of how the internship and your interactions with the student are progressing.

INSTRUCTIONS FOR RECORDING

- Download the app: Voice Record Pro.
- Select 'REC'.
    - A quality overview will appear; no adjustments are necessary.
    - Select 'REC' again.
- Start recording:
    - Begin by stating your name, the day, and the time of the recording. This helps me locate the fragment later.
    - Document and explore the event/situation you want to share (see guiding questions above).
- Stop the recording:
    - Select 'STOP'.
- Rename the file: Rename the file to "Location – Date – Student".
    - If you want to re-record, repeat the steps above.
- Send the recording:
    - Choose 'send by email'.
    - Select 'audio' and then 'email attachment'.
    - Send to hannelore.vanderkloot@kuleuven.be.
    - If the file is too large, choose 'download link'.
