# Supplementary material for: The Power of Students: Using Positioning Theory and Frame Analysis to Explore Power Dynamics in Mentoring Relationships
Source: Perspect Med Educ. 2025 May 28;14(1):328–38. doi: 10.5334/pme.1662 (PMC12124276; doi:10.5334/pme.1662)
Supplement: Supplementary file 2. — Appendix 2. Interview guides for mentors. [file pme-14-1-1662-s2.pdf]

Appendix 1 – Interview Guides for Mentors

Interview Guide 1 – Before Internship

Introduction

Research overview

Before we begin the interview, I would like to briefly remind you of the purpose of this research. The goal is to gain insights into how the relationship between mentors and mentees develops during the internship. We have already scheduled several moments to observe your interactions, for which I am very grateful. I also hope you are willing to share your experiences through audio diaries on days when I cannot observe. In the two interim interviews, we will delve deeper into these aspects together. Do you have any questions about this?

Purpose of this Interview

I propose we start the interview. This first interview aims to get to know you better in your role as a mentor, map your collaboration with the university and colleagues in practice, and explore your expectations for the upcoming internship with (student's name).

Consent for Recording

Do you agree to have this interview recorded? This will ensure I don't have to take extensive notes while you speak. This allows me to focus fully on what you say and give my complete attention to the conversation. You can request to pause or stop the interview or recording at any time.

Start Recording
Now we can begin the interview.

A. Biographical Questions

I'd like to start with some questions about your previous experiences as a mentor and how you perceive yourself in this role.

Experience

A.10 How did you become a mentor?

> **A.11** How long have you been a mentor?

A.20 To what extent did you feel prepared for your role as a mentor?

> **A.21** Who or what helped you in this regard?
> **A.22** Are you currently receiving support in your role as a mentor? This can include both formal and informal support.
> **A.23** What types of support are available to you?
> **A.24** Whose support, or what kind of support, has been most meaningful to you?

A.30 What are your experiences as a mentor in the context of general practice?

Approach of mentoring

Now, I'd like to delve deeper into how you approach your role as a mentor.

A.40 How would you describe yourself as a mentor using three adjectives? For example: "I am a ... mentor."

A.41 Why did you choose these adjectives?

A.42 (For mentors with more experience): If I had asked you this question five years ago, would you have chosen the same adjectives? Why or why not?

A.50 When do you look back on an internship with a sense of satisfaction?

A.51 What needs to happen (or not happen) for you to feel that way?

- **A.511** What is your role and responsibility as a mentor in this?
- **A.512** What is the student's role and responsibility?

A.60 What do you think students expect from you during their internship?

A.61 What is your opinion of these expectations?

A.62 How do you concretely try to meet these expectations?

A.63 To what extent do you think you generally meet these expectations?

- **A.631** What makes you think so?

A.70 How would you describe an "ideal intern"?

A.71 What does this person do well or not well?

A.72 What should the intern have achieved by the end of the internship?

A.80 What do you hope students will learn from you during their internship?

A.81 How do you support their learning?

A.82 What is the most important message you hope the student takes away at the end of the internship?

A.90 What is the most important piece of advice you would give to colleagues just starting out as mentors with their first intern?

B. Support and Collaboration in Mentorship

Next, I would like to discuss your collaboration with the university.

Collaboration with the University

B.10 How would you describe your collaboration with the university?

B.11 What guidelines for mentorship do you receive from the university?

B.12 To what extent is there contact between you and the university supervisor (before, during, and after the internship)?

- **B.121** How often does the university supervisor visit during the internship?

- **B.122** What types of interactions are there (e.g., evaluations, administrative)?

B.13 How would you describe the collaboration between you and the university supervisor?

- **B.131** What do you see as the most important task or responsibility of the university supervisor? (For the student/for the mentor)

Support within the workplace

B.20 Now, I'd like to ask a few questions about support within the workplace.

B.21 How supported do you feel by others in the general practice in your role as a mentor?

- **B.211** How does this support manifest?

B.22 Do you ever discuss with colleagues in the practice how to approach internship mentoring?

C. Expectations

In this final section, I'd like to explore your specific expectations for the upcoming internship with (student's name). Specifically, I'm interested in your expectations of the student and your mentoring relationship.

The Student

C.10 How well do you know the student you will be supervising?

C.11 Have you already met or spoken with them?

- **C.111** How did you experience that conversation or those conversations?

The Mentoring Relationship

Finally, I'd like to ask some questions about your mentoring relationship.

C.20 Soon, there will be a consultation where the student works independently.

C.21 What do you see as your role as a mentor in this situation?

C.22 Will there be a debrief afterward? Why or why not?

- **C.221** What does this debrief look like?
- **C.222** When does it take place?
- **C.223** What is the goal of the debrief?

C.23 To what extent do you plan to be present during the student's consultations?

C.24 How often will the student conduct consultations independently?

C.25 Are there specific expectations or guidelines for this from the university? If so, what are they?

C.30 What other activities do you plan to include in the student's supervision, beyond discussing consultations or patient cases? Why?

Conclusion

This brings us to the end of the interview. Are there any additional points you'd like to mention?

Thank you for your openness and time.

Discussion of the future plans

- New observation date
- Explanation of the audio diary
- Date for the next interview

Interview Guide 2 – During Internship

Introduction

Purpose
Thank you for making time for the second interview. I greatly appreciate it. We will start by reflecting on the observations and audio diaries from the past weeks. Then, we'll revisit the expectations you shared during the first interview to see how the internship is progressing in relation to those. Lastly, I'd like to look ahead to the remainder of the internship.

Consent for Recording

Do you agree to have this interview recorded again? You can request to pause or stop the interview or recording at any time.

Start Recording

Part 1 – Respondent-Specific Section: Reflecting on Observations and Audio Diaries

To start, I'd like to reflect on a few notable moments from the past weeks. You have highlighted some of these in the audio diaries, and I'd like to discuss them further. I've also noted a few things during my observations that I'd like to revisit with you.

(Refer to specific moments in separate documents per person.)

Part 2 – Reflecting on Expectations and the Collaboration

Now, I'd like to revisit the expectations you shared for this internship and the student during the first interview. I'm curious to hear how the internship is progressing compared to what you initially expected.

The Student

B.10 During the first interview, you described the "ideal intern" as...

B.11 Based on your experiences over the past weeks, would you adjust this description?

- **B.111** What other aspects might you add that weren't mentioned before?

B.12 To what extent does the student meet your description of an ideal intern?
B.13 To what extent do you expect the student to develop further toward becoming the ideal intern you envision?

- **B.121** What needs to happen for that to occur?

B.20 If you were to rate the student's performance today on a scale of 1 to 10, what score would you give?

B.21 Why this score?

B.22 Why not a 4? Why not a 7? Why not a 9? *(depending on the response)*

B.23 What can you do as a mentor to help the student move from their current score to a 9?

- **B.231** (Building on the previous answer): What strategies have you used so far to support the student's learning?
- **B.232** Is this approach different from the way you've worked up until now?

B.24 I'd now like to explicitly ask you to evaluate the student's performance. To what extent have you already discussed your assessment with the student? How do you think they perceive how they're doing?

Collaboration

B.30 How is your collaboration with the student progressing so far?

B.31 Which aspects of the collaboration are going well?

- **B.311** What is your responsibility in this, and what is the student's?

B.32 Which aspects need attention or adjustment?

- **B.321** What is your role and responsibility in this, and what is the student's?

B.40 Over the past weeks, you've had several supervision conversations with the student (before, during, or after consultations, as well as a midterm evaluation).

B.41 Can you describe a conversation where you thought, "This went really well"?

- Follow-up questions:
  - What made this conversation so successful? (Was it about the collaboration, the student's development, etc.?)
  - Do you think the student experienced this conversation in the same way? Why or why not?

B.42 Can you describe a conversation that was more challenging?

- Follow-up questions:
  - What made this conversation more difficult? (Was it about the collaboration, the student's development, etc.?)
  - Do you think the student experienced this conversation in the same way? Why or why not?

B.50 What do you think (student's name) expects from you as a mentor?

B.51 To what extent do you think you're meeting the student's expectations? *(Probe whether they perceive this as an issue.)*

B.60 What compliment would you give yourself for your guidance of this student?

B.70 Where do you see areas for improvement or points of attention for yourself?

B.71 What could help you with this? (e.g., peer coaching, support from the university...)

C. Conclusion

C.10 (Student's name) has X weeks left in the internship. When will you look back on this internship with a sense of satisfaction as a mentor?

This brings us to the end of the interview. Are there any additional points you'd like to mention?

Thank you again for your openness and time.

Discussion of the Plan

- Observations and audio diaries
- Final interview

Interview Guide 3 – After Internship

Introduction

Purpose
Thank you again for making time for this final interview. We will start by reflecting on the past observations and audio diaries. Then, we'll revisit the expectations you shared during the first and second interviews to evaluate how those were realized. Lastly, we'll reflect on the student's evaluation and the collaboration with the university.

Consent for Recording

Do you agree to have this interview recorded again? You can request to pause or stop the interview or recording at any time.

Start Recording

Part 1 – Respondent-Specific Section: Reflection on Observations and Audio Diaries

To begin, I'd like to reflect on a few notable moments from the past weeks. You highlighted some of these moments in the audio diaries, and I'd like to revisit them with you. I've also noted some things during my observations that I'd like to discuss further.

(Refer to specific moments in separate documents per person.)

Part 2 – Reflection on Expectations and Collaboration

Now, I'd like to revisit the expectations you shared throughout the internship and assess how they have been met.

The Student

B.10 In the second interview, you mentioned that the student (did/did not) meet your description of an "ideal intern." Has anything changed over the past few weeks?

> **B.11** How did you experience working with a student who (did/did not) match your description of an ideal intern?
>
> **B.12** To what extent is meeting your description of an "ideal intern" an indicator of a successful evaluation? In other words, if an intern meets your description, does that usually result in high marks?

B.20 In the second interview, you gave the student a score of (X) for their performance during the internship. Has this score changed?

> **B.21** What caused this change, or why did it stay the same? *(Probe: Was it due to the student, your supervision, or a combination?)*
>
> **B.22** Where has the student shown the most growth?

B.30 How would you visualize the student's progress over time on a graph? *(Bring paper for this exercise.)* The x-axis represents time, and the y-axis represents your perception of the student's performance. Feel free to include peaks and valleys. *(Probe further based on the graph.)*

B.31 To what extent does this growth resemble the development of previous interns you've supervised?

B.32 During which moments did the student learn the most? Why do you think that is?

B.33 Where were there missed learning opportunities, and how would you explain them? *(Probe further.)*

B.40 What final tips would you give the student for their future as a general practitioner in training?

Collaboration

B.50 To what extent has the collaboration between you and the student changed over the final weeks of the internship?

B.51 What caused these changes or kept things the same?

B.52 How do you now reflect on the overall collaboration throughout the internship?

B.60 To what extent have the supervision conversations (before, during, and after consultations) remained the same or changed over time?

B.61 How would you explain these changes (or lack thereof)?

B.62 What do you think about these developments?

Part 3 – Student Evaluation

The internship has now ended, and the evaluation has been completed. *(Ask specific questions depending on the student-mentor pair.)*

Part 4 – Collaboration with the University

D.10 To what extent was there contact with the university during the internship?

D.11 How did you experience the collaboration and communication with the university throughout the internship? (Was it too little, too much?)

D.12 At which moments would closer contact with the university have been beneficial?

D.20 Is it correct that internship supervisors are fully responsible for the student's final evaluation?

D.21 How does it feel to be responsible for this final assessment? *(To what extent do you feel capable and confident in this role?)*

D.22 Would you like to receive support from the university in this process? If so, what would that look like?

D.30 To what extent do you find the assignments set by the university valuable for the internship? *(e.g., assignments on communication, cervical cancer prevention, and chronic patient case discussions.)*

D.31 How did you approach these assignments (and their supervision)?

Part 5 – Reflection on the Internship

Now, I'd like to ask some concluding questions. The internship is (almost/fully) complete...

E.10 How do you look back on this internship?

> **E.11** What word best describes this internship for you?
>
> **E.12** How does this student differ from others you've supervised during internships?

E.20 What are you most proud of as a mentor?

E.30 Knowing what you know now, what would you have done differently in your supervision of this internship, and why? *(If necessary, probe further)*: Are there aspects where you feel disappointed or that opportunities were missed? Which ones?

Part 6 – Conclusion

F.10 Before we wrap up, how have you experienced my involvement throughout this process?
F.20 Are there any other expectations you have of me?

This brings us to the end of the interview. Are there any additional points you'd like to mention?

Thank you once again for your openness and time, both now and over the past weeks.
