# Supplementary material for: The Power of Students: Using Positioning Theory and Frame Analysis to Explore Power Dynamics in Mentoring Relationships
Source: Perspect Med Educ. 2025 May 28;14(1):328–38. doi: 10.5334/pme.1662 (PMC12124276; doi:10.5334/pme.1662)
Supplement: Supplementary file 3. — Appendix 3. Interview guides for students. [file pme-14-1-1662-s3.pdf]

Appendix 2 – Interview Guides for Students

Interview guide 1 – Before Internship

Introduction

Research Overview
Before we start the interview, I'd like to briefly remind you of the purpose of this research. The goal is to gain insights into how the relationship between mentors and mentees develops during internships. We have already scheduled several moments for me to observe your interactions, for which I'm very grateful. I also hope you're willing to share your experiences through audio diaries on days when I'm not observing. During the two interim interviews, we can delve deeper into these topics together. Do you have any questions about this?

Purpose of the Interview
Let's begin the interview. This first interview is aimed at getting to know you better as a medical student and intern, mapping your collaboration with and supervision by the university and the practice, and exploring your expectations for the upcoming internship with (mentor's name).

Consent for Recording
Do you agree to have this interview recorded? This ensures I don't have to write down everything you say. This way, I can focus fully on what you're saying, giving my full attention to the conversation. You can ask to pause or stop the interview or recording at any time.

Start Recording
Now we can begin the interview.

A. Biographical Questions

A.10 It's your 1st/2nd/... day of the internship. How is it going so far?

Experience

We'll discuss the current internship shortly. First, let's focus on your previous experiences as an intern.

A.20 This isn't your first internship...

> **A.21** How do you look back on your previous internships?

- **A.211** In which specialty were they?
- **A.212** Did they go well or not? Why?
- **A.213** What role did the mentor play in that?

A.22 To what extent do these experiences influence how you plan to approach this internship, particularly your collaboration with the mentor? *(see link to Part B)*

A.30 In previous internships, which mentors stood out the most to you, both in their supervision and as physicians?

> **A.31** ... in a positive way? You don't need to mention names, but how would you describe this person (e.g., using adjectives)?

- **A.311** What made their supervision so positive?
- **A.312** What did you learn from this mentor?
- **A.313** How did this mentor help you?

A.32 ... in a negative way? Again, no names are needed, but how would you describe this person?

- **A.321** What made their supervision negative?
- **A.322** What would you want to see done differently in supervision?

A.40 How would you describe an “ideal mentor”?

A.41 What does this person do well or not well?

A.50 Thinking back on previous internships, who else provided you with guidance or support outside of the supervisor and formal mentor?

A.51 What types of support were these? *(e.g., professional, personal, social...)*

A.52 What made this guidance or support so meaningful?

Internship

Next, I’d like to briefly delve into who you are as an intern and future general practitioner.

A.60 How would you describe yourself as an intern, using three adjectives?

A.61 Why did you choose these adjectives?

A.70 What were you hoping to learn during each internship?

A.80 When do you look back on an internship with a sense of satisfaction?

A.81 What needs to happen (or not happen) for you to feel that way?

- **A.811** What is your role and responsibility as a student in achieving this?
- **A.812** What is the role and responsibility of the mentor?

B. Expectations

In this final section, I’d like to explore your specific expectations for this upcoming internship with (mentor’s name). I’m particularly interested in your expectations regarding the internship itself, the mentor, and your relationship with them.

Expectations for the Internship

B.10 Continuing from the previous question: When will you look back on this internship with satisfaction? *(looking for internship goals)*

B.11 What needs to happen (or not happen) for this to occur?

B.12 What is your responsibility in achieving this?

- **B.121** What is the mentor’s responsibility?

B.20 Did you personally choose to do this general practice internship (adapt if necessary) during this period?

B.21 If yes, what appeals to you about general practice?

B.22 If no, to what extent do you want to participate in this internship?

Expectations for the Mentor

Now let's look at your expectations for the mentor.

B.30 Do you know the mentor who will guide you?

B.31 Have you already met or spoken with them before the start of the internship?

- **B.311** How did you experience that conversation(s)?

B.40 (Following from A.70) You mentioned earlier that you typically hope to learn (paraphrase student's response). What do you specifically hope to learn during this internship? *(repeat only if needed)*

B.41 How do you hope the mentor will help you with this?

B.50 What do you think the mentor expects from you during the internship?

B.51 What do you think of those expectations?

B.52 How do you plan to address those expectations?

Expectations for the Relationship

Lastly, I have a few questions about your relationship with the mentor.

B.60 How do you think the coming weeks will look like?

B.61 Do you expect to conduct consultations independently, for example?

B.70 Ideally, how often would you have discussions with the mentor?

- **B.71** How would you structure these discussions?
- **B.72** What would you like to achieve during these discussions?

B.80 Are there specific guidelines from the university regarding the contact between you and the mentor?

- **B.81** What do these discussions look like?
    - **B.811** When do they usually take place?

C. Support and Supervision by the University

Now we can move to the final part of the interview, which focuses on the support provided by the university.

C.10 How were you prepared for this internship by the university?

- **C.11** Were you given specific guidelines or objectives?
- **C.12** What types of support are provided by the university? *(e.g., return sessions)*

Focus on the University Supervisor

C.20 To what extent is there contact between you and the university supervisor before, during, and after the internship?

- **C.211** How often does the university supervisor visit during the internship?
- **C.212** What types of contact moments are there? *(e.g., evaluations, administrative interactions)*
  C.22 How does the collaboration between you and the university supervisor work?
- **C.221** To what extent has this collaboration been developed?
  C.23 What do you see as the primary task or role of the university supervisor?

D. Conclusion

This brings us to the end of the interview.

D.10 What do you wish for yourself over the coming weeks?

D.20 Are there any additional points you would like to mention?

Thank you for your openness and time.

Discussion of the Plan

- New observation date
- Explanation of the audio diary
- Date for the next interview

Interview Guide 2 – During Internship

Introduction

Purpose
Thank you for making time for the second interview. I really appreciate it. We'll start by reflecting on the past observations and the audio diaries. Then, we'll reconnect with the expectations you shared for this internship during the first interview and evaluate to what extent they have been realized. Finally, I'd like to look ahead to the remainder of the internship.

Consent for Recording

Do you agree to have this interview recorded again? You can ask to pause or stop the interview or recording at any time.

Start Recording

Part 1 – Respondent-Specific Section: Observations and Audio Diaries

To start, I'd like to reflect on a few notable moments from the past weeks. You've highlighted some of these in the audio diaries, which I'd like to discuss further. I've also noted a few things during my observations that I'd like to revisit with you.

(Refer to specific moments in separate documents per person.)

Part 2 – Reflecting on Expectations and Collaboration

Now I'd like to revisit the expectations you expressed for the internship and the mentor during the first interview. I'm curious about how the internship has been progressing compared to what you initially expected.

Internship

B.10 How satisfied are you with your internship so far?

B.20 If you were to rate your performance during the internship on a scale of 1 to 10, what score would you give yourself?

> **B.21** Why this score?
>
> **B.22** What do you think you could still do to move from X to Y?
>
> **B.23** What could the mentor do to help you move from X to Y?
>
> **B.24** What score do you think the mentor would give you?
>
> - **B.241** Why do you think this score would be higher/lower than your own?

Mentor

B.30 During the first interview, you described an "ideal mentor" as… To what extent does this mentor meet that description?

> **B.31** Based on your experiences over the past weeks, would you adjust your description of the ideal mentor?

- Are there any additional aspects you’d add that weren’t mentioned before?

B.32 To what extent does this mentor meet your adjusted description of the ideal mentor?

B.33 To what extent do you expect the mentor to grow toward your description of the ideal mentor?

- **B.331** What would need to happen for that to occur?
- **B.332** To what extent is it possible to discuss your assessment of the mentor with them?

B.40 If you were to evaluate the mentor in their role, what score out of 10 would you give them?

B.41 Why this score? *(Explore positive aspects and areas for improvement.)*

Collaboration

B.50 How has your collaboration with the mentor been progressing so far?

B.51 Which aspects of the collaboration are going well?

- **B.511** What is your responsibility in this, and what is the mentor’s?

B.52 Which aspects need attention or possible adjustment?

- **B.521** What is your role and responsibility in this, and what is the mentor’s?

B.60 Over the past weeks, you’ve had various supervision discussions with your mentor (before, during, and after consultations, as well as the midterm evaluation).

B.61 Can you describe a discussion where you thought, “Okay, this is going really well”? *(Follow-up questions:)*

- What made this discussion go so well? *(Was it about the collaboration, your development, etc.?)*
- Do you think the mentor experienced this discussion in the same way? Why or why not?

B.62 Can you describe a discussion that was more challenging?
(Follow-up questions:)

- What made this discussion more difficult? *(Was it about the collaboration, your development, etc.?)*
- Do you think the mentor experienced this discussion in the same way? Why or why not?

B.70 What do you think (mentor’s name) expects from you as an intern?

B.71 To what extent do you think you’re meeting the mentor’s expectations?

B.80 What compliment would you give yourself regarding your collaboration with the mentor?

Part 3 – Conclusion

C.10 There are still about three weeks left in the internship. When will you look back on this internship with a sense of satisfaction?

This brings us to the end of the interview. Are there any additional points you'd like to mention?

Thank you for your openness and time.

Discussion of the Plan

- Observations and audio diaries
- Final interview

Interview Guide 3 – After Internship

Introduction

Purpose
Thank you for making time for this final interview. We'll begin by reflecting on the past observations and audio diaries. Then, we'll reconnect with the expectations you shared for this internship during the first interview and evaluate to what extent they have been realized. Finally, we'll reflect on the overall internship experience.

Consent for Recording

Do you agree to have this interview recorded again? You can ask to pause or stop the interview or recording at any time.

Start Recording

Part 1 – Respondent-Specific Section: Observations and Audio Diaries

To start, I'd like to reflect on a few notable moments from the past weeks. You've highlighted some of these moments in the audio diaries, which I'd like to revisit. I've also noted a few things during my observations that I'd like to discuss further with you.

(Refer to specific moments in separate documents per person.)

Part 2 – Reflecting on Expectations and Collaboration

Now I'd like to return one last time to the expectations you expressed for the internship and the mentor during the first interview. I'm curious to hear how the internship unfolded compared to your expectations.

Mentor

B.10 In the second interview, you mentioned that the mentor (did/did not – specify per mentoring case) meet your description of an "ideal mentor." Has anything changed over the past weeks?

> **B.11** How did you experience being supervised by someone who (did/did not – specify per mentoring case) match your description of an ideal mentor?
>
> **B.12** To what extent does having an "ideal mentor" contribute to having an "ideal internship"? *(Does an ideal mentor help you reach your full learning potential for the internship?)*

B.20 In the second interview, you gave the mentor a score for their supervision. Has that score changed now?

> **B.21** What caused this change, or why did it stay the same?
>
> **B.22** Where has the mentor been most helpful to you?
>
> **B.23** What tips would you give the mentor for guiding future internships?

Collaboration

B.30 To what extent has your collaboration with the mentor changed during the final weeks of the internship?

> **B.31** What contributed to these changes or kept things the same?

> **B.32** How do you now reflect on your overall collaboration throughout the internship?

B.40 To what extent have the supervision discussions remained the same or changed over time?

> **B.41** How would you explain these changes (or lack thereof)?

> **B.42** What is your opinion on these developments?

Part 3 – Reflection on the Internship

The internship is now fully complete.

C.10 How do you look back on this internship?

> **C.11** What word would you choose to describe this internship? Why?

> **C.12** How does this mentoring experience differ from others you've had?

C.20 How would you visualize your learning progress over time on a graph? *(Bring paper for this exercise.)* The x-axis represents time, and the y-axis represents your perception of your own performance. Feel free to include peaks and valleys. *(Follow up with probing questions!)*

- Where did you learn the most, and why do you think that was?
- Where were there missed learning opportunities, and how would you explain them?

C.30 To what extent are you satisfied with what you've learned?

> **C.31** *(Depending on respondent-specific content)* Are you also satisfied with the mentor's evaluation of you?

C.40 What are you most proud of?

C.50 Are there any aspects you feel disappointed about or opportunities you think were missed? Which ones?

C.60 When you talk with fellow students about their internship experiences, what aspects of theirs do you envy? And, conversely, what aspects of yours might they envy?

Part 4 – The University

Now I'd like to ask a few questions about your interactions with the university during the internship.

D.10 To what extent was there contact with the university during the internship?

> **D.11** How did you experience the collaboration and contact with the university? *(Was it too little, too much?)*

> **D.12** At what moments would closer contact with the university have been helpful?

D.20 Is it correct that supervisors are solely responsible for your evaluation? What do you think about this?

> **D.21** Would you like to receive evaluations from the university as well? If so, what would that look like?

D.30 To what extent do you find the assignments given by the university valuable for the internship? *(e.g., assignments on communication, cervical cancer prevention, and chronic patient case discussions.)*

> **D.31** How did you approach these assignments?

Part 5 – Conclusion

F.10 Before we finish, how did you experience my involvement throughout this process?

F.20 Are there any additional expectations you have of me?

This brings us to the end of the interview. Are there any additional points you'd like to mention?

Thank you once again for your openness and time, both now and throughout the past weeks.
